# Anton’s Syndrome following Callosal Disconnection

**DOI:** 10.1155/2007/306075

**Published:** 2007-08-22

**Authors:** J. Abutalebi, C. Arcari, M. A. Rocca, P. Rossi, M. Comola, G. C. Comi, M. Rovaris, M. Filippi

**Affiliations:** ^1^Centre of Cognitive NeuroscienceUniversity Vita-Salute San RaffaeleMilanItaly; ^2^Interdisciplinary Center for Cognitive StudiesUniversity of PotsdamGermany; ^3^Neuroimaging Research UnitUniversity Vita-Salute San Raffaele and Scientific Institute San RaffaeleMilanItaly; ^4^Department of NeurologyUniversity Vita-Salute San Raffaele and Scientific Institute San RaffaeleMilanItaly

**Keywords:** Cortical blindness, Anton’s syndrome, disconnection, SPECT, fMRI

## Abstract

Anosognosia for cortical blindness, also called Anton’s syndrome, is a rare neurological disorder usually following bilateral lesions to occipital cortices. Neuropsychological, morphological and functional neuroimaging (SPECT and fMRI) findings are reported in a patient who incurred Anton’s syndrome after an ischaemic lesion confined to the left occipital lobe involving the corpus callosum. The present case study suggests that Anton's syndrome may also follow from lesions disconnecting the occipital cortices.

## 1. Introduction

Cortical blindness refers to visual loss in the presence of intact pregeniculate visual pathways. The common pathologic component is bilateral ischemia of the occipital cortex, either as a result of a local event such as embolic or thrombotic occlusion of the posterior cerebral arteries, or more commonly as a result of a global process such as hypoxic or ischaemic encephalopathy [1]. Transient basilar artery insufficiency has been suggested as a major pathological mechanism for this occipital predilection [9] since the occipital cortex is particularly sensitive to systemic hypoxia because of its relatively distal location from the central cerebral vasculature [11].

Visual anosognosia, also called Anton's syndrome, is a rare complication of cortical blindness [2] generally resulting from damage to visual association cortices. Patients suffering from Anton's syndrome deny blindness and [1] typically offer excuses when confronted with the loss of vision ("there is not sufficient light to see") and may endanger themselves "proving" that their vision is intact (e.g., bumping into walls and obvious impediments). As for cortical blindness, also Anton's syndrome is usually associated with bilateral destruction of occipital cortices [7,12].

To the best of our knowledge, Anton's syndrome for cortical blindness has not been reported after focal unilateral lesions. Here we present Anton's syndrome following functional disconnection of the occipital convexities due to a callosal transfer deficit.

## 2. Case report

A 32-year old man suffered an intra-ventricular haemorrhage due to rupture of an aneurysm of the left posterior cerebral artery (PCA). The patient underwent four-vessel angiography and embolization of

ISSN 0953-4180/07/$17.00 © 2007 – IOS Press and the authors. All rights reserved the aneurysm. Ten days later a widespread vasospasm complicated his clinical course and the patient subsequently showed transcortical sensory aphasia and cortical blindness. On clinical examination, pupillary light reflexes and extraocular movements were intact whereas functions dependent on the visual cortex such as the blink response to threat were bilaterally absent. The fundoscopic examination was entirely normal. MR scanning revealed an ischemic lesion confined to the mesial part of the left occipital lobe involving the splenium of the corpus callosum (Fig. 1). VEPs in the acute phase showed increased latency for stimuli within the right visual field. Repeated VEPs in the lesion phase (2 months post-onset) entailed preserved bilateral latencies to flash stimulation.

Clinically, aphasia completely recovered within days while visual deficits persisted. Visual deficits consisted of complete blindness. He was even not able to distinguish between darkness and daylight. During the experimental neuropsychological evaluation aimed at detecting perceptive and associative visual deficits (see Table 1) a complete blindness was observed in almost all visual tasks. The detecting, positioning, pursuing, description and recognizing entirely failed in both visual fields regardless whether the visually presented stimuli were moving or not, of animate or inanimate nature, big or small or in distinct colours. The few correct responses that were observed (i.e. naming correctly a visually presented colour) were casual responses. All experimental sessions were videotaped with permission of the patient.

Visual loss was complicated by a strong denial of blindness. Indeed, he was convinced of good visual performance. Even when bumping into chairs or walls when walking around in our department, he insisted that his visual abilities were perfect. When informed about his blindness by his parents and care-givers he felt in a subjective state of confusion characterized by anxiety, sadness, and aggressive and opposing behaviours.

His visual imagery was unimpaired except for topographic imagery for well-known places or route descriptions (see Table 1).

A general neuropsychological evaluation was carried out in the lesion phase with parts of standardized batteries that do not require visual processing. Preserved linguistic, mnestic, logical and praxic skills were found. His verbal IQ (WAIS) was 109. However, tactile anomia confined to his left hand was encountered.

Tc-99m-ECD SPECT and fMRI were both performed in the lesion phase (2 months post-onset). SPECT imaging revealed hypoperfusion of the left middle and inferior occipital gyrus, the left cuneus/precuneus, the left fusiform gyrus and the posterior parts of the left middle temporal and lingual gyrus. When lowering the threshold to $p < 0.01$ a relative hypoperfusion of the right occipital lobe was also observed (Fig. 1). An fMRI study was performed that consisted of two tasks: 1) projection, through a mirror system of a high-contrast reversing checkerboard pattern alternated with a fixation cross; and 2) tactile stimulation (finger tapping) of the palm of the right hand. During the first task, significant activations were observed in the left insula, in several regions of the left temporal lobe (ex-

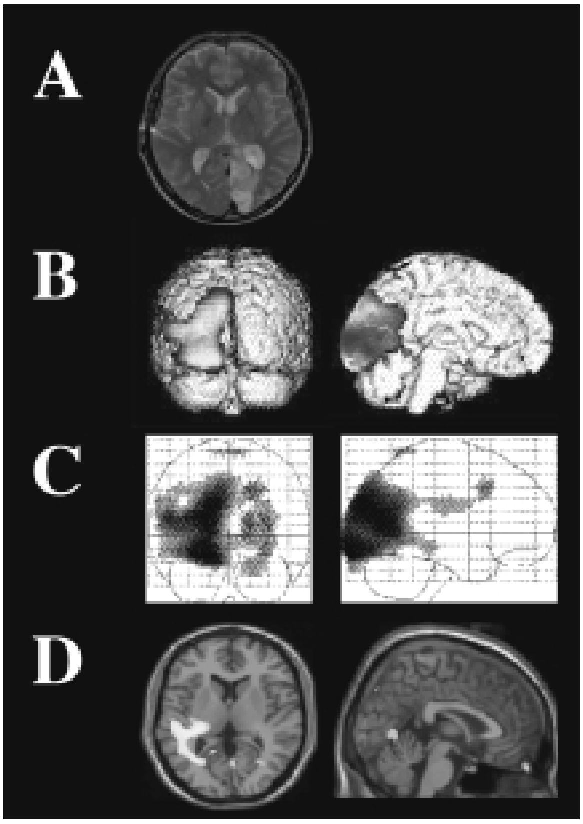

Fig. 1. Conventional MRI and functional neuroimaging of a patient with anosognosia for cortical blindness after a left occipital ischemic lesion also involving the splenium of the corpus callosum, as shown on the axial T2-weighted image (**A**). Tc-99m-ECD SPECT imaging revealed hypoperfusion of the left middle and inferior occipital gyrus, the left cuneus/precuneus, the left fusiform and the posterior parts of the left middle temporal gyrus and lingual gyrus at a threshold of $p < 0.001$ (**B**). At a lower threshold ($p < 0.01$), a relative hypoperfusion of the right occipital lobe was also seen (**C**). FMRI related brain activations during task 1 are superimposed on a high resolution T1-weighted image in (**D**). Brain activations were found in the left posterior insula, in several regions of the left temporal lobe, in the right cuneus and, bilaterally, in the anterior lingual gyrus. All images, except the conventional MRI image in (**A**), are presented as $L \rightarrow L$; $R \rightarrow R$.

Table 1
Patient's performance on the experimental neuropsychological investigation carried out to assess perceptive and associative visual deficits. The material used for the experimental investigation was derived from the BORB (Birmingham Object Recognition Battery; Riddoch MJ, & Humphreys GW, 1993) and from the unpublished dissertation of one of the authors (CA)

| | |
|---|---|
| **• Perceptive/Associative Tasks** | |
| Colour Denomination | 1/10 |
| Object Recognition | 1/10 |
| Image Recognition | 0/10 |
| Recognition of Moving Objects* | 1/20 |
| **• Visual Imagery Tasks** | |
| Description of the Dome Square in Milan | inadequate |
| Description of local routes | inadequate |
| Description of letter shapes | 20/20 |
| Description of letter graphic forms | 15/15 |
| Comparison of letter graphic forms | 14/14 |
| Description of common symbols | 18/20 |
| Description of car brand symbols | 11/12 |
| Description of traffic code symbols | 10/10 |
| Comparison of animal sizes | 28/30 |
| Description of animal ears (straight or pendant?) | 17/20 |
| Description of animal tails (short or long?) | 20/20 |
| Description of animal legs (short or long?) | 27/30 |
| Comparison of animal distinctive features | 19/22 |
| Description of object sizes | 25/30 |
| Description of object features | 26/30 |
| Description of object widths | 29/30 |
| Comparison of object distinctive features | 19/21 |
| Comparison of spatial relationship between object parts | |
| – Metric | 14/15 |
| – Topologic | 15/15 |
| Description of typical colors of objects, vegetables, and animals | 45/50 |

*Administered for both visual fields.

cluding MT/V5), in the right cuneus and V1 and bilaterally in the anterior part of the lingual gyrus (Fig. 1). Significant deactivations were observed in the left precuneus and V1, and in MT/V5, bilaterally. During tactile stimulation, significant activations were observed in the left primary and secondary sensorimotor cortices, in perilesional regions and in the right MT/V5. No deactivations were detected.

## 3. Discussion

The patient developed Anton's syndrome following damage to the left occipital lobe and the splenium of the corpus callosum.

Preservation of the right primary visual cortex and some remnant parts of the left primary visual cortex was demonstrated with conventional MRI and fMRI revealing activations seen during task 1. These findings may explain the normal pattern observed during the VEPs examination. Normal VEPs in patients with cortical blindness have been frequently reported and this might be mediated by extrageniculo-calcarine connections between the optic nerve and the secondary visual cortex [10]. However, conscious visual perception is preserved only when a critical amount of area 17 is spared [4].

fMRI findings revealed preserved function of V1 in the right hemisphere and reduced V1 activation in the damaged hemisphere in task1. Moreover, several additional areas of the ventral stream of visual stimuli processing located in the temporal lobe were recruited in the damaged hemisphere. It is worth noting that, contrary to previous studies performed on subjects with V1 lesions [6], we also observed a deactivation of MT/V5 complex, bilaterally. Although this region receives major input from the primary visual cortex, bilateral destruction of V1 does not suppress its visual responsiveness [6]. Its reduced activation in our patient might be related to a disrupted interhemispheric connection between V1 and MT/V5 complex. During task 2, we observed the activation of left perilesional areas and of the MT/V5 complex in the healthy hemisphere. The activation of the visual areas during tactile or auditory stimulation has been demonstrated by several studies performed on blind people and has been considered a consequence of tactile-visual cross-modal plasticity [3]. The different behaviour of V1 and MT/V5 in the two hemispheres is likely related not only to the V1 lesion of the left hemisphere, but also to the involvement of the splenium with the consequent disconnection of the visual areas.

Anton's syndrome is usually reported after bilateral lesions to the occipital lobes with extensive damage to associative visual areas [7,12]. However, awareness of illness is generally ascribed to right hemispheric functions [8]. One major hypothesis regarding the pathogenesis of anosognosia is indeed the disconnection of the right hemisphere from its left sided counterpart (see [8] for a review). The present case underlines that impaired conscious vision complicated by anosognosia, that is, Anton's syndrome, may be also caused by damage to the left occipital lobe and the splenium of the corpus callosum disconnecting the former from its right-sided counterpart as shown by SPECT and fMRI imaging. Tactile anomia confined to the patient's left hand further confirms the callosal disconnection.

The present report emphasizes that functional neuroimaging techniques are suitable to investigate and explain neurological deficits with unusual lesion locations. For instance, in 'unusual' cases of cortical blindness, i.e., following unilateral lesions, SPECT imaging was successful in reporting bilateral abnormalities [5]. Here we provided evidence that also an 'unusual' case of Anton's syndrome was associated with bilateral abnormalities as detected with SPECT and fMRI.

**All authors declare that there are no competing interests.**
